# Differential flow improvements after valve replacements in bicuspid aortic valve disease: a cardiovascular magnetic resonance assessment

**DOI:** 10.1186/s12968-018-0431-5

**Published:** 2018-02-08

**Authors:** Malenka M. Bissell, Margaret Loudon, Aaron T. Hess, Victoria Stoll, Elizabeth Orchard, Stefan Neubauer, Saul G. Myerson

**Affiliations:** 1 Division of Cardiovascular Medicine, Radcliffe Department of Medicine, University of Oxford Centre for Clinical Magnetic Resonance Research (OCMR), Headley Way, Oxford, OX3 9DU UK; 2 Department of Cardiology, Oxford University Hospitals NHS Trust, Oxford, UK

**Keywords:** Magnetic resonance imaging, Aortic disease, Bicuspid aortic valve, Aortic valve replacement, 4D flow

## Abstract

**Background:**

Abnormal aortic flow patterns in bicuspid aortic valve disease (BAV) may be partly responsible for the associated aortic dilation. Aortic valve replacement (AVR) may normalize flow patterns and potentially slow the concomitant aortic dilation. We therefore sought to examine differences in flow patterns post AVR.

**Methods:**

Ninety participants underwent 4D flow cardiovascular magnetic resonance: 30 BAV patients with prior AVR (11 mechanical, 10 bioprosthetic, 9 Ross procedure), 30 BAV patients with a native aortic valve and 30 healthy subjects.

**Results:**

The majority of subjects with mechanical AVR or Ross showed normal flow pattern (73% and 67% respectively) with near normal rotational flow values (7.2 ± 3.9 and 10.6 ± 10.5 mm^2^/ms respectively vs 3.8 ± 3.1 mm^2^/s for healthy subjects; both *p* > 0.05); and reduced in-plane wall shear stress (0.19 ± 0.13 N/m^2^ for mechanical AVR vs. 0.40 ± 0.28 N/m^2^ for native BAV, *p* < 0.05). In contrast, all subjects with a bioprosthetic AVR had abnormal flow patterns (mainly marked right-handed helical flow), with comparable rotational flow values to native BAV (20.7 ± 8.8 mm^2^/ms and 26.6 ± 16.6 mm^2^/ms respectively, *p* > 0.05), and a similar pattern for wall shear stress. Data before and after AVR (*n* = 16) supported these findings: mechanical AVR showed a significant reduction in rotational flow (30.4 ± 16.3 → 7.3 ± 4.1 mm^2^/ms; *p* < 0.05) and in-plane wall shear stress (0.47 ± 0.20 → 0.20 ± 0.13 N/m^2^; *p* < 0.05), whereas these parameters remained similar in the bioprosthetic AVR group.

**Conclusions:**

Abnormal flow patterns in BAV disease tend to normalize after mechanical AVR or Ross procedure, in contrast to the remnant abnormal flow pattern after bioprosthetic AVR. This may in part explain different aortic growth rates post AVR in BAV observed in the literature, but requires confirmation in a prospective study.

## Background

Bicuspid aortic valve disease (BAV) is the most common congenital heart disease (1–2% of the population) and is associated with ascending aortic dilation in up to 80% of cases [1]. While traditionally the BAV aortopathy has been described as an intrinsic aortopathy, recent advances in cardiovascular magnetic resonance (CMR) have suggested that the flow pattern in the ascending aorta may be a major contributor to the aortopathy in BAV [2–5]. 4D flow CMR allows visualization and quantification of the blood flow in the aorta in a 3D image. Using this technique we have recently shown that the inherent restricted leaflet opening of BAV, even when functioning normally, causes an asymmetrical, off center flow jet which hits the ascending aortic wall, leading to marked rotational (helical) flow [4]. This abnormally high rotational flow is associated with increased ascending aortic diameters. Part of the proposed mechanism includes increased wall shear stress, which is a surrogate marker for the friction the blood flow exerts onto the aortic wall [4]. The concept that increased wall shear stress contributes to the BAV aortopathy is further supported by a recent study which showed that histopathological changes (such as reduced elastin) in excised aortas from BAV patients only occurred in areas with high wall shear stress but not in areas with normal or low wall shear stress, as assessed with 4D flow CMR prior to aortic resection [6].

Patients with a BAV suffer from valve disease at a younger age than patients with trileaflet aortic valves and the majority of BAV patients will require aortic valve replacement (AVR) in their lifetime [7]. Given the likely pathophysiological effects of the abnormal helical flow on aortic dilation in BAV disease, examining the flow patterns in the proximal aorta after AVR may provide novel insights. A recent pilot study examined these in mainly trileaflet aortic valve disease [8], and suggested that different AVR types may result in different flow patterns. To date however, no study has assessed the impact of AVR alone on the flow abnormalities in specifically BAV. We therefore hypothesized that AVR may favorably alter flow patterns in the ascending aorta in patients with a BAV. We also sought to examine in this pilot study the flow patterns after different AVR types (bioprosthetic versus mechanical) to determine if these differ.

## Methods

### Patient recruitment

We prospectively enrolled 105 participants recruited from cardiology clinics and CMR lists between January 2011 and July 2015 in a cross-sectional cohort study. This included 45 BAV patients either awaiting or having previously undergone AVR surgery. We excluded any BAV patients with other complex heart disease such as atrioventricular septal defect, hypoplastic left heart syndrome and aortic coarctation. From the 45 patients, we excluded 7 patients from the main analysis who underwent concomitant aortic root replacement, 5 patients who underwent aortic valve repair and 3 patients who had CMR contraindications after AVR. Prosthetic valve types in the remaining 30 patients were mechanical bileaflet (*n* = 11), bioprosthetic (*n* = 10) or a Ross procedure (*n* = 9). The 11 patients with mechanical AVR (AVR-mechanical) had bileaflet tilting disc type in all cases, either an ATS Medical Manufacturing Company (Minneapolis, Minnesota, USA) valve (23-25 mm), *n* = 5; or an ON-X ACE (CryoLife, Incorporated, Kennesaw, Georgia, USA) aortic valve (21-27 mm), *n* = 6. Valve models in the bioprosthetic aortic valve replacement group (AVR-tissue) were Perimount Magna Ease (Edwards Lifesciences Corporation, Irvine, California, USA) (23-27 mm), *n* = 4; Trifecta (Abbot, St. Jude Medical, Abbott Park, Illinois, USA) (23-27 mm), n = 4; Hancock II porcine (Medtronic, Minneapolis, Minnesota, USA) (27 mm), *n* = 1 and Mitroflow (LivaNova, London, England) pericardial (21 mm), *n* = 1. The Ross group had a longer time interval between operation (often in childhood) and imaging. We compared the valve replacement groups with 30 age- and sex-matched healthy subject cohort and 30 un-operated bicuspid aortic valve patients matched to the post-operative peak velocity values of the AVR group. All of these control patients participated in our initial cohort study [4]. A sub-group of 16 BAV patients also had CMR assessment both pre- and post-operatively, allowing comparison of flow patterns before and after AVR. Thirteen of these patients participated in our initial cohort study [4] prior to their valve replacement. The study complies with the declaration of Helsinki and was approved by the West Berkshire ethics committee. All participants gave written informed consent.

### Cardiovascular magnetic resonance acquisition

Each subject underwent a CMR scan on a 3 Tesla system (Trio, Siemens Healthineers, Erlangen, Germany) for anatomical and 4D flow assessment using a 32-channel cardiac surface coil. All images were electrocardiogram (ECG)-gated. Balanced steady-state free-precession (bSSFP) cine sequences acquired during a single breath-hold were used for aortic dimension measurements at the level of the pulmonary arteries and for left ventricular (LV) volume assessment [9]. The velocity across the aortic valve was measured using through-plane phase contrast velocity mapping in an image slice placed perpendicular to the ascending aorta, just above the valve tips (at the vena contracta). Commercial CMR42 software (Circle Cardiovascular Imaging Inc., Calgary, Canada) was used for analysis of standard anatomical and velocity parameters. Aortic diameters were measured from inner edge to inner edge at end-diastole.

### 4D flow assessment

Flow-sensitive gradient-echo pulse sequence CMR was used to characterize and quantify 4D flow hemodynamics in the thoracic aorta. Datasets were acquired with prospective ECG-gating during free-breathing, using a respiratory navigator. The image acquisition volume was in an oblique sagittal plane encompassing the whole thoracic aorta. Sequence parameters: echo time 2.5 ms, repetition time 5.1 ms, flip angle 7° (15° if Multihance (gadobenate dimeglumine, Bracco Milan, Italy) contrast agent was used to improve image quality in patients with a peak velocity > 3 m/s), voxel size 1.9–2.0 × 1.5–1.7 × 2.0–2.2 mm^3^, temporal resolution 40 ms. The velocity encoding range was determined using the lowest non-aliasing velocity on scout measurements (healthy subject cohort 1–1.5 m/s; BAV patients 1.5–4.5 m/s). Dataset processing, calculation of flow angle, flow displacement, rotational flow and wall shear stress calculation were conducted with custom software in Matlab version R2010a and R2015a (The MathWorks Inc., Natick, Massachusetts, USA) and EnSight Version 10.02(d) (CEI Inc., Apex, North Carolina, USA) as described previously [10–13]. All measures were averaged over peak systole (one time frame before and three after peak systolic flow) to mitigate measurement noise.

**Fig. 1:**
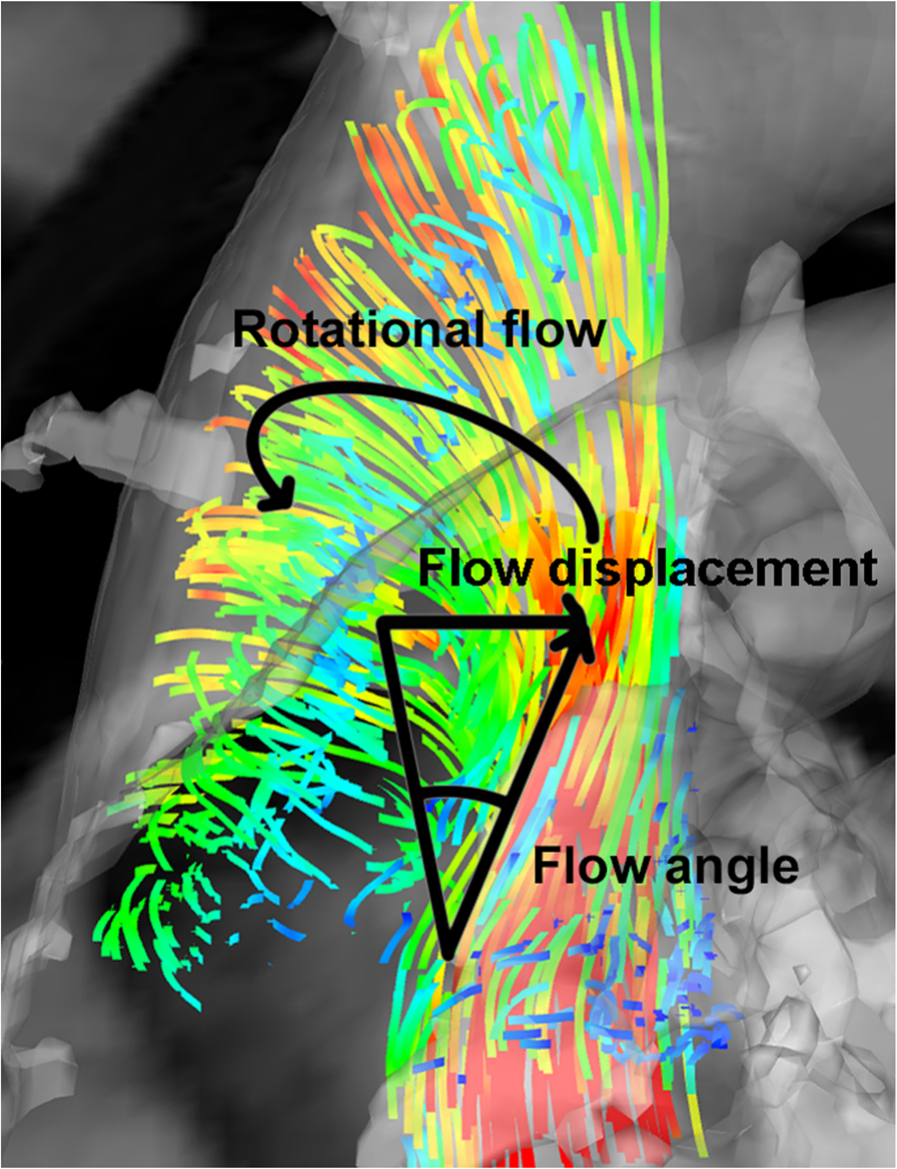
Depiction of flow angle, flow displacement and rotational flow

Helical flow (Fig. 1 and Table 1) was quantified using the rotational component of flow (which is the integral of vorticity with respect to the cross-sectional area of the aorta), and categorized as defined previously [4], with normal-helical flow between − 5 and 11 mm^2^/s, abnormal right-handed rotational flow > 11 mm^2^/s, and abnormal left-handed rotational flow < − 5 mm^2^/s. To enable comparable statistical analysis, absolute rotational flow values were used to assess the amount of rotational (helical) flow irrespective of the orientation of the rotation. Complex flow was defined as a lack of an observable coherent rotational (helical) flow pattern.

**Table 1:** 4D Flow parameter

Flow angle	The angle of deviation from the center of the aortic lumen
Flow displacement	The distance of flow jet deviation from the center of the aortic lumen indexed to ascending aortic diameter
Rotational flow	The blood flow circling within the aortic plane
WSS_circavg_	Circumferentially averaged systolic wall shear stress - surrogate marker for the friction the blood flow exerts onto the aortic wall
In-plane wall shear stress	(the ‘rotational’ component) exerted by the blood circling within the plane along the aortic wall
Through-plane wall shear stress	Exerted by the blood flowing through the vessel.

The flow angle (Fig. 1 and Table 1) represents the angle between the line perpendicular to the short axis analysis plane of the aorta and the instantaneous mean flow vector at peak systole [14]. Flow displacement (Fig. 1 and Table 1) was calculated as the distance from the vessel center line centroid to the velocity-weighted centroid (the center of the flow jet) as described by Sigovan et al. [10] and normalized to aortic diameters. Both flow angle and displacement calculation assume that the “normal” flow is along the midline which is not the case after mechanical valve replacement as a mechanical valve usually leads to two off center jets. Therefore flow angle and flow displacement may be less meaningful parameters in this group.

Wall shear stress (Table 1) was calculated using the 3-dimensional flow vector and magnitude data and measured in eight anatomical positions around the aortic wall as well as the circumferentially averaged systolic wall shear stress (WSS_circavg_). This combined the vector magnitude of both through-plane and in-plane (rotational) wall shear stress, as described previously [3, 4].

### Statistical analysis

SPSS Statistics Version 24 (International Business Machines Corporation, Armonk, New York, USA) was used for statistical analysis. Data were tested for normal distribution with the Kolmogorov-Smirnov test. Normally distributed data were analyzed using one-way ANOVA with post-hoc Games-Howell analysis for multiple group comparison. The paired student t-test was used for comparison of individuals’ data before and after valve replacement, while unpaired (independent) t-test was used for between-group comparisons. A *p*-value < 0.05 was considered significant. Independent t-test was used for group comparison.

## Results

### General demographics and valve characterization

General demographics of the different BAV groups and controls are summarized in Tables 2 and 3. As expected, the AVR-tissue group were older (mean of 58 years) and the Ross procedure (Ross) group were younger (mean of 24 years) compared to the AVR-mechanical group (mean of 42 years). The native BAV group, the AVR-mechanical and AVR-tissue all had significantly larger ascending aortic diameters compared to healthy volunteers, whereas only the Ross group’s ascending aortic diameters were similar to the healthy subject group. By exclusion criterion, no patient had an additional (repaired) coarctation of the aorta or a hypoplastic arch. Peak velocity was similar in all patient groups and only the Ross group showed significant regurgitation post AVR (mean regurgitant fraction 22.8%). Mild patient-prosthesis mismatch occurred in only one patient, who was in the AVR-mechanical group (effective orifice area 0.8 cm^2^/m^2^).

**Table 2:** Ascending aortic flow pattern parameters by aortic valve replacement subgroup

	Healthy Subjects	BAV- native	AVR-mechanical	AVR-tissue	Ross	
Total number (male)	30 (23)	30 (23)	11 (10)	10 (9)	9 (8)	
Time after operation in months (range)			6 (3–14)	9 (1–37)	126 (1–268)	
Indication for AVR:						
Aortic stenosis			4	7	8	
Aortic regurgitation			5	1	1	
Both			2	2		
Fusion pattern pre-AVR:						
RL-BAV			7	5	Data	
RN-BAV			2	5	unavailable	
Others			2			
Flow pattern post-AVR:						
Normal flow	30	5 (17%)	8 (73%)	0	6 (67%)	
Right-handed flow		21 (70%)	2 (18%)	8 (80%)	2 (22%)	
Left-handed flow		1 (3%)	1 (9%)	1 (10%)	1 (11%)	
Complex flow		3 (10%)	0	1 (10%)		

Values are mean ± standard deviation except where indicated; BAV = bicuspid aortic valve; AVR = aortic valve replacement; RL-BAV = right-left coronary cusp fusion pattern bicuspid aortic valve, RN-BAV = right-non-coronary cusp fusion pattern bicuspid aortic valve

**Table 3:** Ascending aortic flow pattern parameters by aortic valve replacement subgroup

	Healthy Subjects *n* = 30	BAV- native *n* = 30	AVR-mechanical *n* = 11	AVR-tissue *n* = 10	Ross *n* = 9	ANOVA
Age in years	43 ± 17	42 ± 16	42 ± 12	58 ± 11	24 ± 12	*p* < 0.001
Systolic flow angle (°)	7.5 ± 5.3	25.6 ± 13.3*	16.5 ± 9.6	16.2 ± 8.5	9.8 ± 4.9†	p < 0.001
Normalised flow displacement	0.038 ± 0.036	0.133 ± 0.072*	0.083 ± 0.076	0.163 ± 0.064*	0.096 ± 0.067	p < 0.001
Absolute rotational flow (mm^2^/ms)	3.8 ± 3.1	26.6 ± 16.6*	7.2 ± 3.9†	20.7 ± 8.8*	10.6 ± 10.5†	p < 0.001
Mean systolic WSS_circavg_ (N/m^2^)	0.58 ± 0.15	0.77 ± 0.23*	0.67 ± 0.20	0.68 ± 0.20	0.80 ± 0.35	*p* = 0.012
Absolute systolic in-plane (rotational) WSS (N/m^2^)	0.07 ± 0.06	0.40 ± 0.28*	0.19 ± 0.13†	0.38 ± 0.19*	0.21 ± 0.17	p < 0.001
Max systolic through-plane WSS (N/m^2^)	0.76 ± 0.21	0.95 ± 0.44	0.91 ± 0.38	0.78 ± 0.215	1.03 ± 0.65	*p* = 0.206
RA-LP systolic WSS_circavg_ (N/m^2^)	−0.12 ± 0.13	0.22 ± 0.31*	0.27 ± 0.22*	0.36 ± 0.26*	0.24 ± 0.67	p < 0.001
Peak velocity (m/s)	–	2.3 ± 0.8	2.1 ± 0.5	2.1 ± 0.3	1.9 ± 0.8	*p* = 0.387
Ascending aortic diameter (mm)	28.6 ± 4.3	35.0 ± 6.7*	35.9 ± 4.4*	37.9 ± 5.2*	30.4 ± 8.3	p < 0.001
Diameter at sino-tubuar junction (mm)	27.9 ± 3.6	32.4 ± 6.4*	31.7 ± 7.8	32.9 ± 3.3	31.0 ± 6.0	*p* = 0.003
Diameter at sinuses (mm)	30.8 ± 3.5	32.9 ± 5.8	34.1 ± 6.7 (pre-AVR)	32.1 ± 2.3 (pre-AVR)	35.9 ± 5.7	*p* = 0.068
Aortic regurgitant fraction (%)	–	8.8 ± 9.9	3.7 ± 2.8	5.8 ± 7.5	22.8 ± 20.6†	*p* = 0.002
Left ventricular ejection fraction (%)	–	68 ± 6	67 ± 15	70 ± 8	65 ± 10	*p* = 0.687

Values are mean ± standard deviation except where indicated; * = *p* < 0.05 compared to healthy volunteers; † = *p* < 0.05 compared to native BAV; BAV = bicuspid aortic valve; AVR = aortic valve replacement; WSS = wall shear stress; WSS_circavg_ = circumferentially averaged wall shear stress; RA-LP = right anterior – left posterior

### Flow patterns after aortic valve replacement

**Fig. 2:**
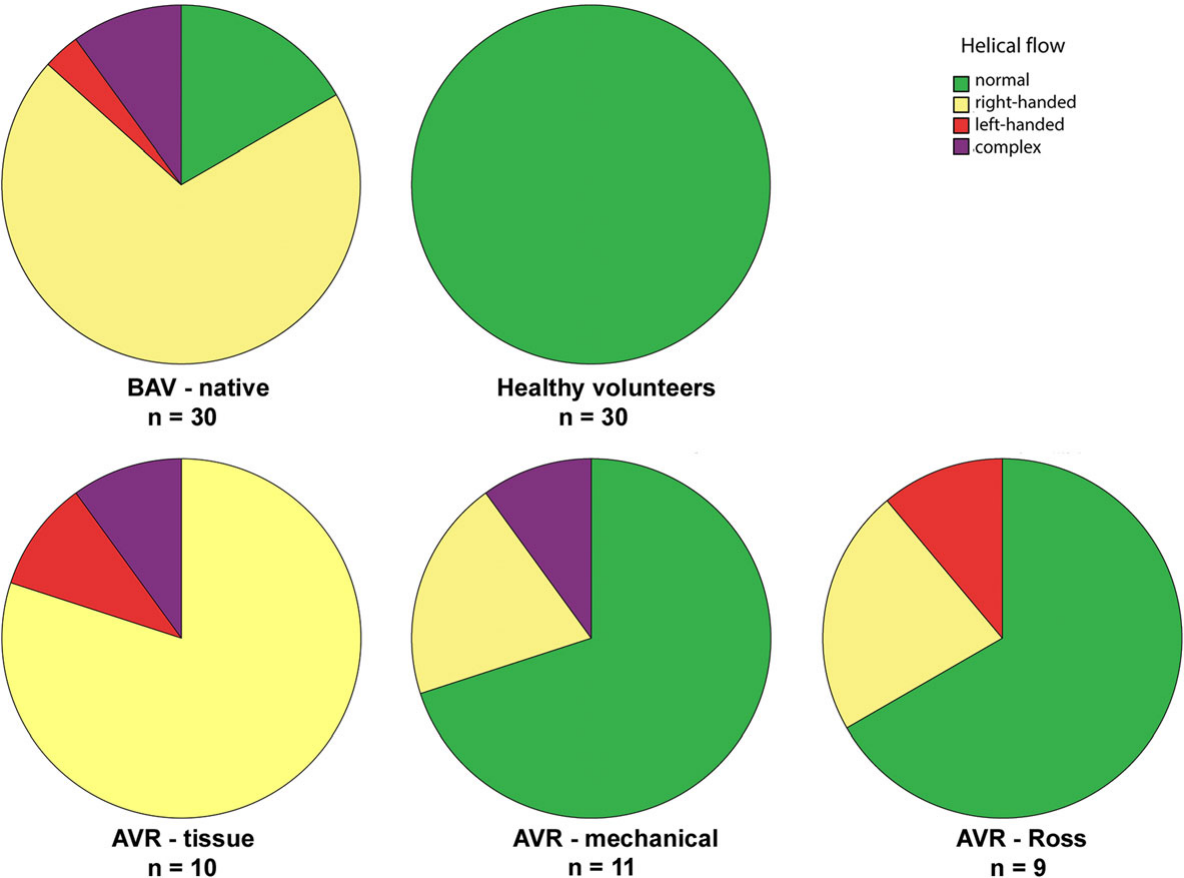
Ascending aortic flow patterns after three types of aortic valve replacement (AVR), compared to un-operated bicuspid aortic valve disease (BAV) and healthy subjects

**Fig. 3:**
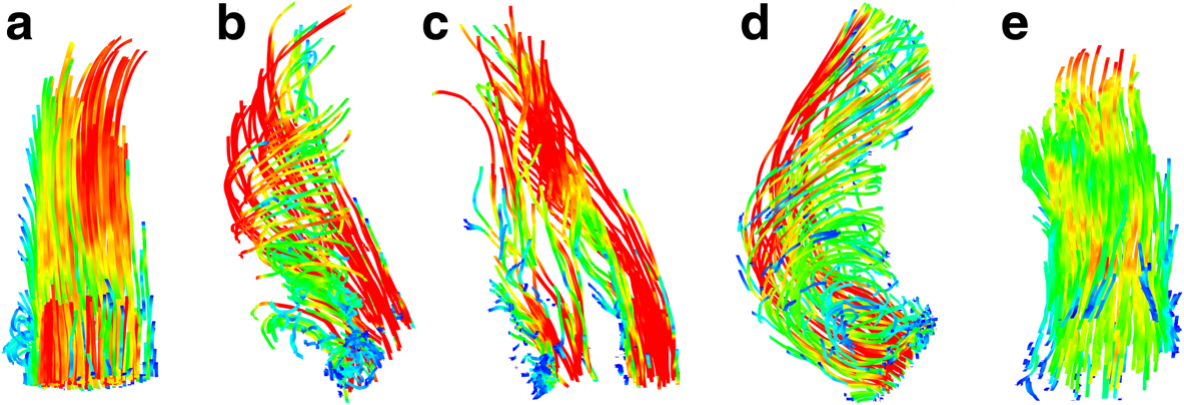
Typical ascending aortic flow patterns after aortic valve replacement (AVR); **a** – healthy subject with a laminar flow pattern; **b** – native bicuspid aortic valve disease with a right-handed helical flow pattern; **c** – AVR-mechanical with 2 laminar jets; **d** – AVR-tissue with a right-handed helical flow pattern; **e** – AVR-Ross with a laminar flow pattern

The distribution of flow patterns is shown in Fig. 2. The majority of BAV patients with a mechanical AVR (73%) and those who had a previous Ross procedure (67%) showed normal aortic flow patterns compared to benchmark patients with a native BAV, in whom only a minority had normal flow patterns (17%). In contrast, the AVR-tissue group demonstrated abnormal helical flow patterns in all patients (Fig. 3).

**Fig. 4:**
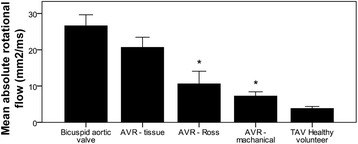
Mean rotational flow – comparison of the different aortic valve replacement groups; * = *p* < 0.05 compared to native un-operated bicuspid aortic valve disease

In keeping with these results, quantitative measures of rotational flow were significantly increased in the BAV-native and AVR-tissue groups (mean values 26.6 and 20.7 mm^2^/s respectively) compared to healthy subjects (3.8 mm^2^/s, Fig. 4). In contrast, in the AVR-mechanical and Ross group the degree of rotational flow was significantly lower and close to normal.

### Flow angle and flow displacement after aortic valve replacement

The flow angle (the angle of deviation from the center of the aortic lumen; Fig. 1) was significantly increased in the native BAV group (25.6°) vs healthy controls (7.5°), *p* < 0.05; Table 3. The AVR-mechanical and AVR-tissue groups both had similar, moderately increased, values (means 16.5° and 16.2° respectively) although these were not significantly different from the healthy controls. The Ross group had normal flow angle values (mean 9.8°).

Flow displacement is the ratio of the distance the flow jet deviates from the center of the aortic lumen, indexed to ascending aortic diameter at the level of the pulmonary artery (Fig. 1). This was also significantly increased in the native BAV and AVR-tissue group (0.133 and 0.163 respectively) compared to the healthy subject group (0.038), *p* < 0.05 (Table 3). The AVR-mechanical and Ross showed a trend towards higher values compared to the healthy cohort group.

### Wall shear stress after aortic valve replacement

Wall shear stress is a surrogate marker for the friction the blood flow exerts onto the aortic wall. The overall wall shear stress, described as the circumferentially averaged systolic wall shear stress (WSS_circavg_), was slightly higher than controls in all three AVR groups (Table 3) but did not reach statistical significance. As expected, it was increased in the BAV-native group compared to healthy subjects (*p* < 0.05). However, wall shear stress consists of two components: The in-plane wall shear stress (the ‘rotational’ component) exerted by the blood circling within the plane along the aortic wall, and the through-plane wall shear stress exerted by the blood flowing through the vessel. When examining these components separately, in-plane wall shear stress was significantly higher in the AVR-tissue and BAV-native groups (0.40 and 0.38 N/m^2^) compared to healthy volunteers (0.07 N/m^2^, p < 0.05), in keeping with the higher degree of rotational flow in these groups, but numbers were small in the AVR-tissue group. In-plane systolic wall shear stress was significantly *lower* in the AVR-mechanical group (0.19 N/m^2^, *p* < 0.05), with similar values in the Ross group. Through-plane wall shear stress (exerted by blood flowing along the long axis of the aorta) was similar across all groups.

**Fig. 5:**
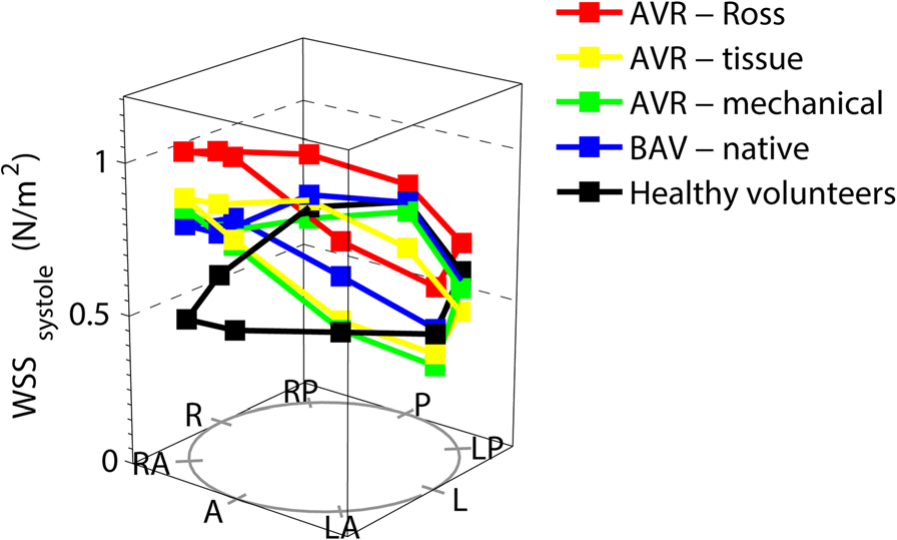
Systolic wall shear stress (WSS): The anatomical positions are shown at the bottom of the Fig. A = anterior (outer curvature); LA = left anterior, L = left, LP = left posterior, P = posterior, RP = right posterior, R = right, RA = right anterior; the height of the dots indicates the wall shear stress value

The BAV-native and all three AVR groups had the highest wall shear stress values in the right anterior position which corresponds to the outer curvature (Fig. 5). In healthy subjects the wall shear stress was highest in the posterior position, which corresponds to the inner curvature.

### Individual changes in aortic flow after aortic valve replacement

**Fig. 6:**
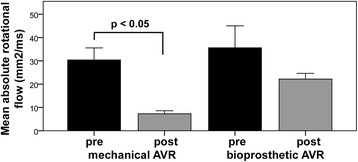
Mean rotational flow – comparison before and after aortic valve replacement (AVR)

Sixteen out of the 30 patients attended a study visit both before and after AVR (10 with mechanical AVR, 6 with a bioprosthetic AVR, Table 4). The AVR-mechanical group all had abnormal flow patterns at baseline (7/10 with right-handed helical flow; 3/10 with complex flow), but after AVR all flow patterns either normalized (7/10) or showed marked reduction in the rotational (helical) component of flow (3/10). As a result the group showed a significant reduction in almost all haemodynamic parameters including mean rotational flow (30.4 ± 16.3 → 7.3 ± 4.1 mm^2^/ms; *p* < 0.05, Fig. 6), WSS_circavg_ (1.02 ± 0.21 → 0.68 ± 0.21 N/m^2^; p < 0.05), as well as both the individual components, in-plane (0.47 ± 0.20 → 0.20 ± 0.13 N/m^2^; p < 0.05) and through-plane wall shear stress (1.43 ± 0.54 → 0.92 ± 0.39 N/m^2^; p < 0.05). The reduction in systolic flow angle and normalized flow displacement did not reach statistical significance (Table 4).

**Table 4:** Comparison ascending aortic flow pattern parameters before and after valve replacement

	AVR-mechanical			AVR-tissue		
	Pre-AVR	Post-AVR	*p*-value	Pre-AVR	Post-AVR	*p*-value
Number	10	10		6	6	
Absolute rotational flow (mm^2^/ms)	30.4 ± 16.3	7.3 ± 4.1	*p* = 0.001	35.6 ± 23.1	22.2 ± 6.0	*p* = 0.18
Systolic WSS_circavg_ (N/m^2^)	1.02 ± 0.21	0.68 ± 0.21	p = 0.001	1.04 ± 0.20	0.70 ± 0.21	*p* = 0.048
Absolute systolic in-plane WSS (N/m^2^)	0.47 ± 0.20	0.20 ± 0.13	p = 0.001	0.56 ± 0.32	0.44 ± 0.19	*p* = 0.41
Max systolic through-plane WSS (N/m^2^)	1.43 ± 0.54	0.92 ± 0.39	p = 0.04	1.47 ± 0.34	0.75 ± 0.21	p = 0.01
RA-LP systolic WSS_circavg_ (N/m^2^)	0.55 ± 0.66	0.28 ± 0.23	*p* = 0.19	0.77 ± 0.29	0.25 ± 0.27	*p* = 0.03
Systolic flow angle (°)	26.5 ± 9.3	17.1 ± 9.8	*p* = 0.77	23.8 ± 9.4	20.2 ± 7.2	p = 0.41
Normalised flow displacement	0.126 ± 0.093	0.069 ± 0.065	p = 0.18	0.166 ± 0.053	0.141 ± 0.068	*p* = 0.54
Peak velocity (m/s)	3.4 ± 1.5	2.0 ± 0.5	*p* = 0.02	3.7 ± 0.8	2.1 ± 0.4	p = 0.001
Regurgitant fraction (%)	38 ± 19	3 ± 2	p < 0.001	12 ± 14	2 ± 2	*p* = 0.21
Left ventricular ejection fraction (%)	71 ± 9	68 ± 16	p = 0.68	75 ± 9	75 ± 5	*p* = 0.91

Values are mean ± standard deviation except where indicated; BAV = bicuspid aortic valve; AVR = aortic valve replacement; WSS = wall shear stress; WSS_circavg_ = circumferentially averaged wall shear stress; RA-LP = right anterior – left posterior

In the AVR-tissue group all patients also had abnormal flow patterns at baseline (4/6 with right-handed helical flow; 2/6 with complex flow). However, all 6 patients had a residual right-handed flow pattern after AVR with similar haemodynamic abnormalities, no statistically significant change in mean rotational flow, normalized flow displacement, systolic flow angle or in-plane wall shear stress. There was a similar reduction in WSS_circavg_ (1.04 ± 0.20 → 0.70 ± 0.21 N/m^2^; p < 0.05), through-plane wall shear stress (1.47 ± 0.34 → 0.75 ± 0.21 N/m^2^; p < 0.05), compared to the AVR-mechanical group. The small size of this group limits the conclusions that can be drawn however.

## Discussion

### Type of ascending aortic valve replacement

**Fig. 7:**
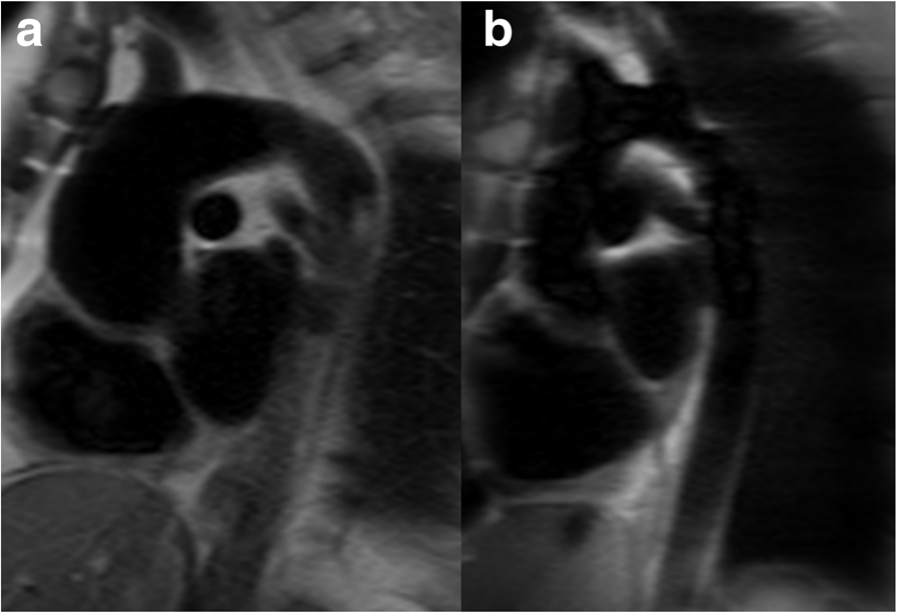
**a** – Patient after bioprosthetic AVR with ascending aortic aortopathy showing the ‘typical’ anteriorly bulging shape; **b** – Patient after Ross procedure showing a normal shaped ascending aorta

Recent advances in CMR imaging have suggested that the flow pattern in the ascending aorta is a major contributor to the aortopathy in BAV [2–5]. The marked helical flow pattern with an increased wall shear stress is thought to play an important role in the pathophysiology. However, very little is known about how flow patterns and wall shear stress are influenced by AVR. Our small study suggests that both the AVR-mechanical and Ross group appeared to have near normalization of flow haemodynamics following AVR, while abnormal helical flows appeared higher in the AVR-tissue group. The findings were similar in the cross-sectional assessments and in those imaged before and after AVR. The numbers in some groups (particularly the AVR-tissue group) were small however and this limits the strength of conclusion. If true, one explanation for this phenomenon may be that a bioprosthetic AVR is inherently associated with increased rotational flow. Abnormal helical flows post-AVR have also been shown in other studies following bioprosthetic AVR for trileaflet aortic valve disease [8, 15–17], supporting the limited data in our study. The pathophysiology for this would be unknown however. We note that all patients with a bioprosthetic AVR imaged pre- and post-surgery had a right-handed helical flow pattern after AVR, even though 2/6 of these patients had had a complex flow pattern pre-operatively, which may suggest that the bioprosthetic valve introduced a right-handed helical flow pattern in those with complex flow preoperatively. An alternative explanation may lie with the age at which the operation occurs. Bioprosthetic AVR is more prevalent in older patients, as was the case in our AVR-tissue group, and bicuspid aortopathy increases with age. At the later age of the bioprosthetic AVR, patients may already exhibit larger aortas with the ‘typical’ anteriorly bulging shape (Fig. 7) [18], and a trileaflet bioprosthetic AVR may not be enough to normalize the flow pattern in this setting. In contrast, the Ross procedure is normally undertaken in childhood before much of the aortopathy has developed, and it is conceivable that replacing the diseased bicuspid aortic valve with a (trileaflet) homograft may normalize the flow at this earlier stage, as observed in our study. A mechanical AVR, on the other hand, introduces a non- physiological flow pattern consisting of two parallel flow jets arising off-center, which our data suggests may reduce the overall rotational flow, even in the presence of a dilated aorta. Confirmation of this would however be required in larger studies.

### Wall shear stress after aortic valve replacement

While the AVR-mechanical group showed a reduction in all wall shear stress components, the AVR-tissue group only showed a statistically significant reduction in through-plane and overall wall shear stress but not in-plane wall shear stress. The reduction in through-plane wall shear stress in both groups is most likely due to the reduction in peak velocities after AVR. The in-plane (rotational) component remained higher in the AVR-tissue group, possibly due to the marked helical flow still seen in these patients. Previous studies have suggested that in-plane wall shear stress is a larger contributor to the aortic dilation in BAV than through-plane wall shear stress [4, 5]. In-plane wall shear stress is negligible in healthy subjects, but significantly increased in BAV patients, even in young patients with normal ascending aortic diameters, normal valve function and resulting normal through-plane wall shear stress, suggesting that the increased in-plane wall shear stress precedes aortic dilation and may be a contributor towards the BAV aortopathy [5].

### Implications of flow on ascending aortic growth

A recent large retrospective study looked at 360 BAV patients after the Ross procedure and showed no difference in aortic growth compared to the general population [19]. The normalization of ascending aortic flow seen in our Ross subgroup may be an explanation for these findings. However, studies of aortic growth after mechanical and bioprosthetic AVR in the literature are inconclusive. All are retrospective, mostly echocardiographic, studies and included either mainly or only mechanical AVR [20–26]. The development of aortic aneurysms (defined as ascending aortic diameter > 4.5 cm) was 10–22% [20, 24], though the need for ascending aorta replacement after 15 years of follow-up remained low at 1–3% [21, 22, 24]. Unsurprisingly, continued aortic dilation was more likely in patients with enlarged aortas at the time of surgery [24]. To date there are few longitudinal studies examining the effect of AVR on aortic growth *rate* and mechanical and bioprosthetic AVR are not separately reported within these studies. While some showed an increased growth rate after AVR (echocardiography [25, 27, 28]; computed tomography (CT) [29]), others showed an overall reduction in aortic growth rate after AVR (echocardiography [23]; echocardiography or CT [30]; CT or CMR [26]). One explanation for these discrepant findings may be that flow profiles differ post mechanical and post bioprosthetic AVR as suggested by the findings in our hypothesis-generating study. Unfortunately no large study has compared ascending aortic growth rate in mechanical vs bioprostheic AVR in BAV, which would be ideal.

Using 4D flow CMR imaging biomarkers as predictors for aortic growth has been examined in one small longitudinal cohort study. In 13 unoperated BAV patients, normalized flow displacement but not wall shear stress correlated with aortic growth [31]. In our cohort, only the AVR-tissue group had significantly increased normalized flow displacement values compared to healthy subjects. The impact of rotational flow has not previously been assessed.

### Limitations

Patient numbers in this study were relatively small and not all patients, particularly the Ross group, had data available both before and after AVR. In the mechanical and bioprosthetic groups, different models of aortic valve prosthesis were used and it is feasible that there are differing flow patterns associated with different valve models. However, both mechanical valve models were of a similar design (bileaflet tilting disc type). In addition, almost all bioprosthetic valves have a similar shaped valve, which is designed to be as close as possible to the native trileaflet valve. All mechanical AVRs were implanted in a R-L orientation in relation to the ascending aorta.

The interval between valve replacement and CMR assessment was up to 3 years in the AVR groups and between 1 month and 22 years in the Ross group, and up to 3 years in other AVR groups. The ages of each AVR group were also significantly different - very young patients tend to undergo a Ross operation, whereas older patients more often opt for a bioprosthetic AVR rather than a mechanical AVR, and age may influence flow patterns through differences in aortic shape and stiffness, which could be an important confounder.

4D flow CMR is a new imaging technique but measures such as wall shear stress have been validated, even though the true wall shear stress is likely to be higher than the measured value, due to limited spatial resolution, as discussed previously [32]. This cross-sectional study is unable to assess the impact of flow normalization on aortic growth rate, for which further longitudinal follow-up will be necessary.

## Conclusion

Abnormal helical flow in BAV disease is significantly reduced after mechanical AVR or Ross procedures. Following bioprosthetic AVR, however, our small group suggested that abnormal helical flow patterns remain similar to un-operated and pre-operative values, but conclusions are limited by the small group size. These findings suggest for the first time that the AVR type may be an influence on aortic flow patterns, but requires confirmation. Whether improved haemodynamics have clinical implications such as a reduction in aortic growth also remains unknown. As ascending aortic growth in BAV is slow, prospective studies would require a long follow-up period. A reasonable alternative to further investigate the potential differences in valve prostheses suggested by our study in a more timely manner, would be larger retrospective studies to compare aortic growth following mechanical and bioprosthetic AVR.

## Abbreviations

AVR: Aortic valve replacement
BAV: Bicuspid aortic valve
bSSFP: Balanced steady-state free-precession
CMR: Cardiovascular magnetic resonance
CT: Computerized tomography
ECG: Electrocardiogram
WSS: Wall shear stress
WSS_circavg_: Circumferentially averaged systolic wall shear stress
